# Influence of scalp block on oncological outcomes of high-grade glioma in adult patients with and without isocitrate dehydrogenase-1 mutation

**DOI:** 10.1038/s41598-021-95851-5

**Published:** 2021-08-13

**Authors:** Chao-Hsien Sung, Fon-Yih Tsuang, Chih-Peng Lin, Kuang-Cheng Chan, Wei-Han Chou, Chun-Yu Wu

**Affiliations:** 1 Department of Anesthesiology, Fu Jen Catholic University Hospital, Fu Jen Catholic University, New Taipei City, Taiwan; 2 Department of Neurosurgery, National Taiwan University Hospital, Taipei, Taiwan; 3 Department of Anesthesiology, National Taiwan University Hospital, No. 7, Chung-Shan S. Road, Taipei, Taiwan

**Keywords:** Outcomes research, Oncology

## Abstract

High-grade gliomas are notorious for a high recurrence rate even after curative resection surgery. Studies regarding the influence of scalp block on high-grade gliomas have been inconclusive, possibly because the condition’s most important genetic mutation profile, namely the isocitrate dehydrogenase 1 *(IDH1*) mutation, had not been analyzed. Therefore, we conducted a single-center study including patients with high-grade glioma who underwent tumor resection between January 2014 and December 2019. Kaplan–Meier survival analysis revealed that scalp block was associated with longer progression-free survival (PFS; 15.17 vs. 10.77 months, *p* = 0.0018), as was the *IDH1* mutation (37.37 vs. 10.90 months, *p* = 0.0149). Multivariate Cox regression analysis revealed that scalp block (hazard ratio: 0.436, 95% confidence interval: 0.236–0.807, *p* = 0.0082), gross total resection (hazard ratio: 0.405, 95% confidence interval: 0.227–0.721, *p* = 0.0021), and *IDH1* mutation (hazard ratio: 0.304, 95% confidence interval: 0.118–0.784, *p* = 0.0138) were associated with better PFS. Our results demonstrate that application of scalp block, regardless of *IDH1* profile, is an independent factor associated with longer PFS for patients with high-grade glioma.

## Introduction

High-grade gliomas are notorious for a high recurrence rate even after gross curative surgical resection, possibly because intense stress and inflammatory response may occur during surgery, inducing immunosuppression that promotes locoregional cancer recurrence^1^. In addition, anesthesia-related factors such as the use of inhalation anesthesia and administration of opioids also induce detrimental immunosuppression^2^. By contrast, regional anesthesia effectively relives surgical stress, reduces inhalational anesthetic and opioid consumption, and has been reported to be beneficial to patients undergoing cancer surgery^1,3^. Therefore, there is increasing interest in investigations of the potential oncological benefits of regional anesthesia for cancer surgery^4^. To date, previous reports have indicated conflicting results regarding the influence of regional anesthesia, namely the scalp block, on postoperative glioma recurrence^5–7^. For instance, we previous reported that scalp block is associated with a prolonged postoperative glioma recurrence^5^. By contrast, another larger cohort revealed negative results^6^. This may be because a tumor’s genetic profile plays a decisive role in the oncological outcomes of patients with glioma^8^, but this has not been analyzed and compared in previous reports of scalp block for glioma resection.

The revised 2016 World Health Organization classification of central nervous system tumors reclassified gliomas on the basis of molecular marker diagnostics combined with classical histological diagnosis^8^. Possibly the foremost molecular markers are mutations in isocitrate dehydrogenase (*IDH*)^9^. Mutations in IDH isozyme 1 (*IDH1*) not only play a crucial role in early tumorigenesis of astrocytomas and oligodendrogliomas but are also the decisive genetic signposts of secondary glioblastoma^10^. Most *IDH1* mutations reported are located at the first or second base of codon 132 and the most frequent is R132H (*IDH1*^R132^^H^)^10^. Patients with glioma and *IDH1*^R132H^ glioblastoma have twice the median overall survival (OS) and more favorable progression-free survival (PFS) than those with the *IDH1* wild type^11,12^. However, occurrence of *IDH1*^R132H^ has not yet been analyzed in the literature regarding scalp block and oncological outcomes. Therefore, in this study, we investigated the influence of scalp block on oncological outcomes in patients undergoing resection surgery for high-grade glioma; a complete *IDH1*^R132H^ genetic profile analysis was performed. To our best knowledge, this presents the first literature of scalp block and the oncological outcomes of high grade glioma with the analysis of *IDH1*^R132H^ mutation.

## Methods

After obtaining the local ethic committee approval, a prospectively maintained database including patients of high-grade glioma undergoing craniotomy for glioma curative resection with complete IDH^R132^ mutation information was used for analysis. Exclusion criteria were (1) age younger than 20 years, because molecular characteristics and prognosis may differ between pediatric and adult patients with high grade glioma^13^, (2) undergoing an awake craniotomy, and (3) receiving chemotherapy or radiotherapy before surgery. Data collected included demographic characteristics, comorbidities, tumor location, tumor size, perioperative red blood transfusion, amount of opioid consumed, presence of the *IDH1*^R132^^H^ mutation, postoperative adjuvant therapies, and anesthetic techniques such as the use of intravenous or inhalation anesthesia and scalp block.

### Surgery and anesthesia

Each patient received general anesthesia that was maintained either intravenously or by inhalation at the attending anesthesiologist’s discretion. Blood transfusion was performed when the patient’s hemoglobin level was less than 9 g/dL. In patients receiving inhalational anesthetics, sevoflurane was used and was kept at 0.5 times the minimum alveolar concentration to minimize interference with intraoperative electrophysiological monitoring^14^. Perioperative steroids were not routinely given because brain lymphoma sometimes resembles glioblastoma on preoperative magnetic resonance images^15^, and steroids administered perioperatively would interfere with pathological examination of the tumor. Application of scalp block was at the anesthesiologist’s discretion and was performed immediately after induction of general anesthesia and before head pinning with a Mayfield head holder; the scalp block regimen was 10 mL of 0.5% levobupivacaine and 1:200,000 epinephrine mixture for each side of the scalp. The effect of the scalp block was achieved by means of local infiltration around targeted nerves, which were the supraorbital, supratrochlear, zygomaticotemporal, auriculotemporal, greater occipital, lesser occipital, and least occipital nerves.

Following surgery, all patients were sent to the same neurosurgical intensive care unit for postoperative care. Postoperative analgesia was achieved with mild to moderate opioids such as tramadol or nalbuphine; nonsteroidal anti-inflammatory drugs were used once the risk of bleeding was considered minimal by the surgeons.

### Statistical analysis

PFS was defined as the interval between the date of surgery and the date of first evidence of tumor recurrence, which was based on postoperative magnetic resonance imaging once every 3 months, as is recommended by guidelines followed by many hospitals worldwide^16^. OS was defined as the interval between the date of surgery and date of death or loss of follow-up. To identify the targeted PFS improvement for at least one follow-up interval (3 months), 90 patients would be needed for statistical analysis with a power of 0.8 and *α* = 0.05.

Fisher’s exact test or the chi-square test was employed to analyze dichotomous data, Student’s *t*-test was used for normally distributed continuous data, and the Mann–Whitney *U* test was used for nonparametric ordinal data. PFS and OS are presented as the median (95% confidence interval [CI]) and were calculated using Kaplan–Meier survival analysis with the log-rank test to compare survival curves between groups. Univariate and multivariate Cox proportional hazards regression analyses were used to determine the effect of several risk factors on PFS and OS. A *p* < 0.05 was considered significant.

In our institute, the complete information regarding the IDH^R132^ mutation was routinely reported in postoperative pathological records since 2014. Because the incidence of high grade glioma was approximately only one per 100,000^17^, it is difficult for patient enrollment. Therefore, this study included high grade glioma patients enrolled in our previous study, which include our institutional glioma patients during 2010 to 2017^18^, to achieve a sufficient study power. Accordingly, a parallel multivariate Cox regression analysis without overlapping was also performed to investigate the influence of scalp blocks on the oncological outcomes of patients with high grade glioma. Statistical analysis was conducted using IBM SPSS Statistics for Windows, version 21.0 (IBM Corp., Armonk, NY, USA) and MedCalc Statistical Software version 18.2.1 (MedCalc Software bvba, Ostend, Belgium).

### Ethics statement

This study was approved by the Research Ethics Committee of National Taiwan University Hospital (approval No.201911066RIND, approval date: December 27, 2019), and the informed consent requirement was waived by the committee. The patients’ identifying information was omitted during analysis. This study adheres to the applicable EQUATOR guidelines.

## Results

### Baseline characteristics

**Figure 1:**
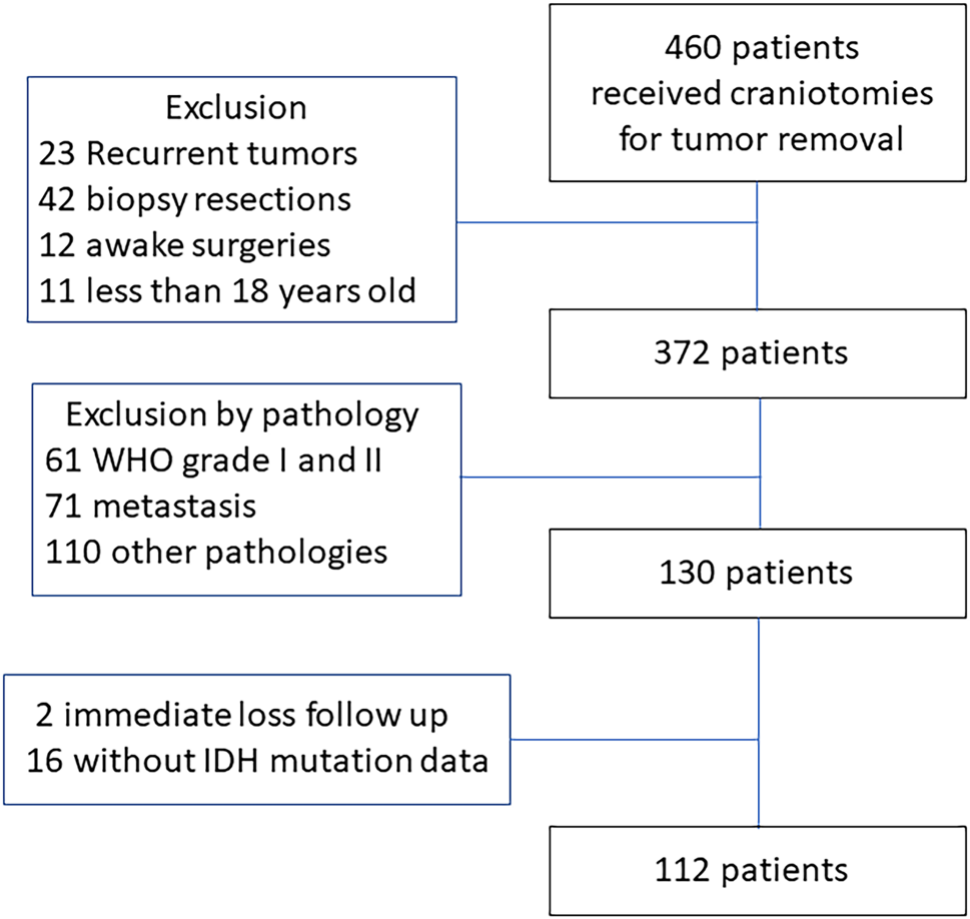
Patient inclusion.

In total, 460 patients underwent craniotomy for tumor resection between January 1, 2014, and December 31, 2019; 112 were finally included in the analysis (Fig. 1). Seventeen patients were revealed to carry the *IDH1* ^R132H^ mutation (15.2%). The median PFS and OS of all patients were 12.40 (8.63–15.17) and 36.83 (22.50–61.13) months, respectively. The 1-year PFS rate of all patients was 50.9%, whereas the median follow-up interval was 15.88 (14.33–18.79) months.

Baseline characteristics between the patients receiving and not receiving scalp block were comparable (Table 1). In our institute, scalp block is often combined with total intravenous general anesthesia (*p* < 0.0001). Lower intraoperative fentanyl consumption was observed in the scalp block group (median fentanyl consumption: 250 [150–300] vs. 300 [200–350] mcg in scalp block vs. non-scalp-block groups, respectively; *p* = 0.0384).

**Table 1:** Characteristics of patients who received scalp block and those who did not receive scalp block.

	No scalp block (N = 58)	Scalp block (N = 54)	p value
Age(years), mean ± SD	59.2 ± 14.6	55.0 ± 15.1	0.1328
Male (%)	29 (50.0)	29 (53.7)	0.7096
Body mass index (kg/m^2^), mean ± SD	23.94 ± 4.36	24.22 ± 4.32	0.7319
**Co-morbidities (%)**			
Cardiovascular Hypertension Diabetes mellitus Liver and renal diseases	8 (13.8) 16 (27.9) 9 (15.5) 2 (3.4)	10 (18.5) 19 (35.2) 9 (16.7) 2 (3.7)	0.6089 0.4200 0.9999 0.9999
**ASA class (%)**			
I II III IV	2 (3.4) 34 (58.6) 21 (36.2) 1 (1.7)	2 (3.7) 32 (59.3) 18 (33.3) 2 (3.7)	0.9227
**WHO grading (%)**			
III IV	13 (22.4) 45 (77.6)	11 (20.4) 43 (79.6)	0.8217
Infratentorial tumor location (%)	4 (6.9)	1 (1.9)	0.3652
Intravenous anesthesia (%)	29 (50.0)	48 (88.9)	< 0.0001
Blood loss (ml), median (interquartile range)	200 (100–400)	200 (100–500)	0.4346
Blood transfusion, red blood (%)	10 (17.2)	10 (18.5)	0.9999
Blood transfusion, non-red blood (%)	2 (3.4)	3 (5.6)	0.6709
Fentanyl usage (mcg), median (interquartile range)	300 (200–350)	250 (150–300)	0.0384
Gross total resection (%)	17 (29.3)	23 (42.6)	0.1695
Anesthesia duration(min), median (interquartile range)	278 (230–335)	295 (265–362)	0.1054
**Surgeon (%)**			
A B C Others	30 (51.7) 15 (25.9) 7 (12.1) 6 (10.3)	33 (61.1) 7 (13.0) 8 (14.8) 6 (11.1)	0.3948
Adjuvant temozolamide (%)	48 (82.8)	42 (77.8)	0.6352
Adjuvant Radiotherapy (%)	51 (87.9)	45 (83.3)	0.5922
IDH1^R132H^ mutation (%)	7 (12.1)	10 (18.5)	0.4322

*ASA* American Society of Anesthesiologists, *WHO* World Health Organization, *IDH* mutation: isohydrate dehydrogenase.

### Scalp block and IDH1 mutation are associated with improved PFS but not OS

**Figure 2:**
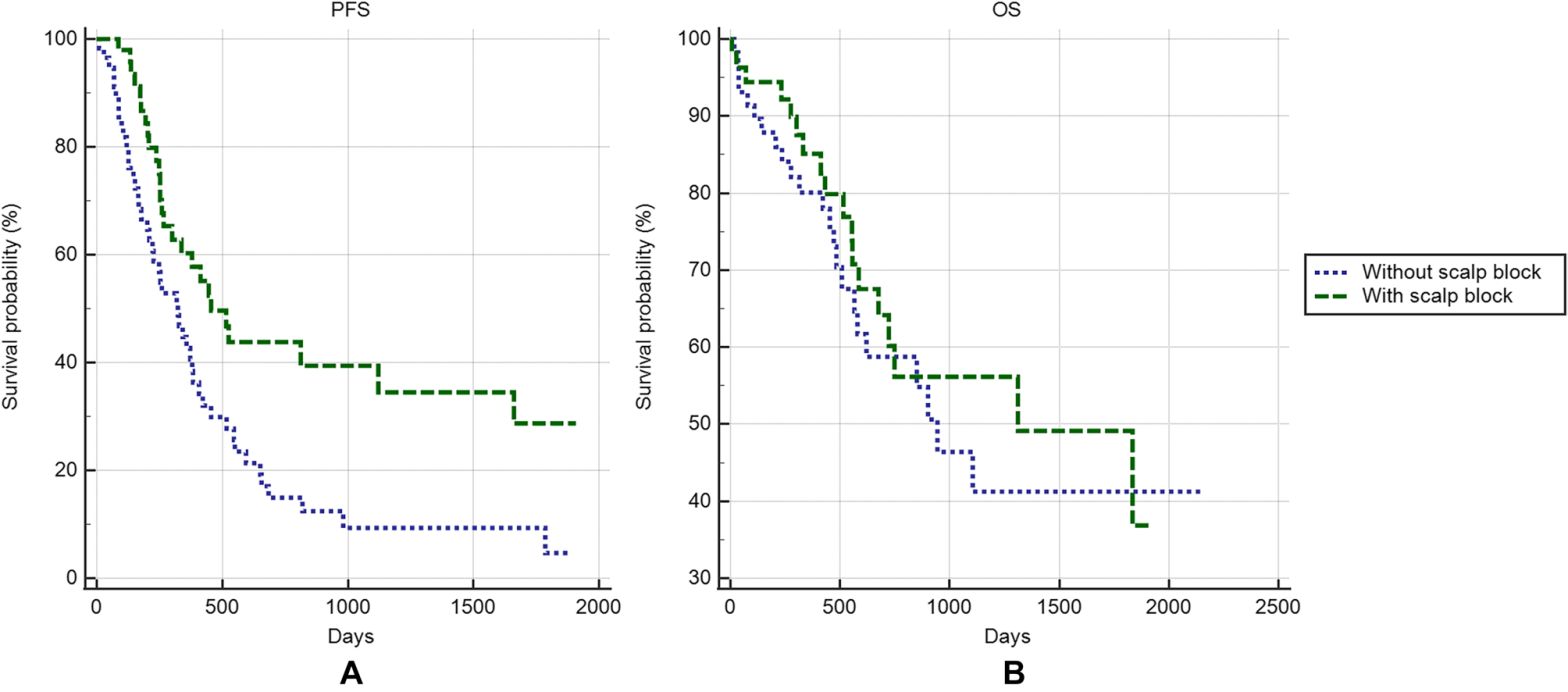
Oncological outcomes between patients who received scalp block and those who did not.

**Figure 3:**
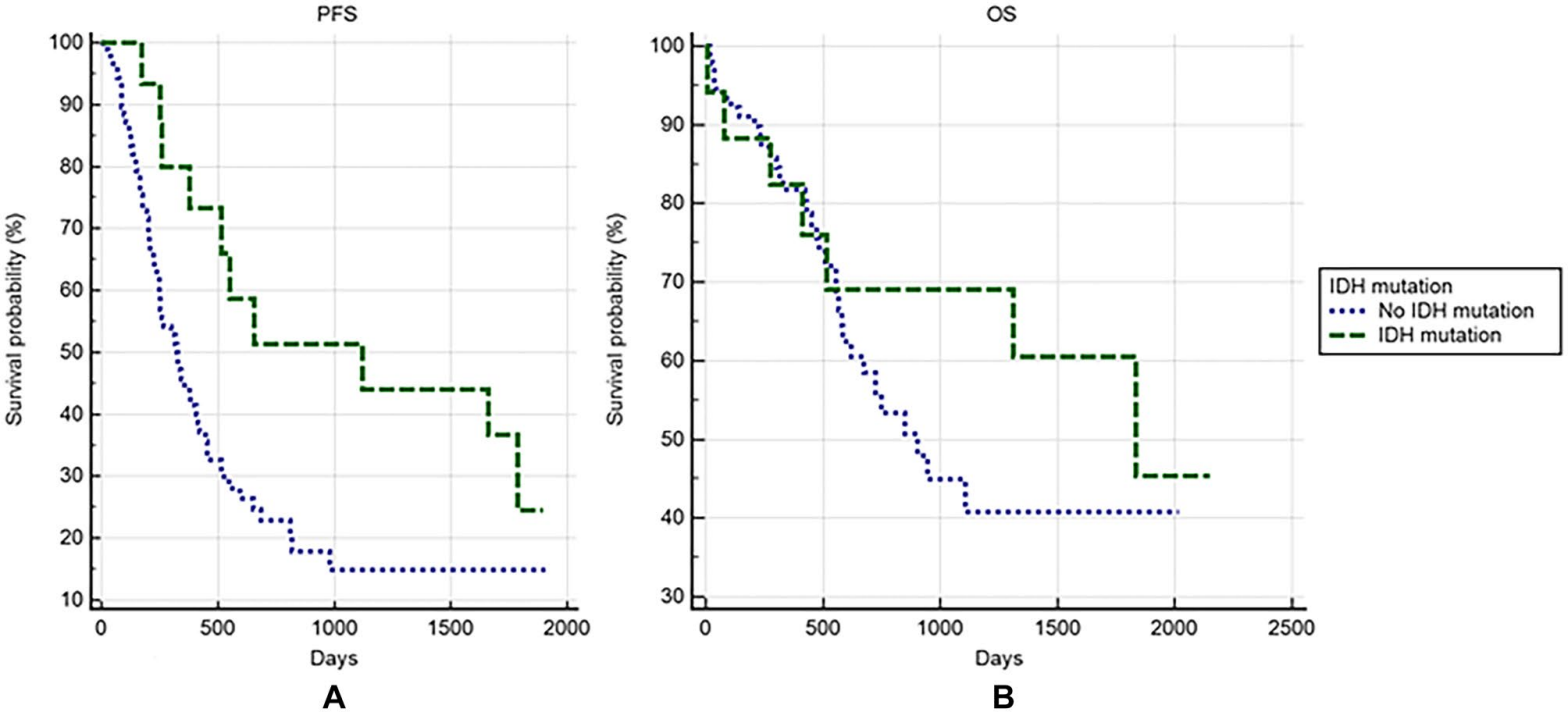
Oncological outcomes between patients with and without IDH1 mutation.

Scalp block was associated with longer PFS (Fig. 2A, median PFS: 15.17 [8.87–37.37] vs. 10.77 [6.97–12.77] months in scalp block vs. non-scalp-block groups, respectively; *p* = 0.0018). However, scalp block was not associated with longer OS (Fig. 2B, median OS: 43.70 [22.50–61.13] vs. 31.47 [18.83–36.83] months in scalp block vs. non-scalp-block groups, respectively; *p* = 0.4929). Although the *IDH1* mutation was associated with longer PFS (Fig. 3A, median PFS: 37.37 [8.63–59.53] vs. 10.90 [8.27–13.77] months in *IDH1* mutation carrier vs. noncarrier groups, respectively; *p* = 0.0149), the *IDH1* mutation was not associated with longer OS (Fig. 3B, p = 0.4021).

### Higher pathology grade and tumor location are additional risk factors associated with reduced OS

Multivariate Cox regression analysis revealed that scalp block (hazard ratio [HR]: 0.436, 95% CI: 0.236–0.807, *p* = 0.0082), gross total resection (HR: 0.405, 95% CI: 0.227–0.721, *p* = 0.0021), and *IDH1* mutation (HR: 0.304, 95% CI: 0.118–0.784, *p* = 0.0138) were associated with longer PFS. World Health Organization grade IV glioma (HR: 2.363, 95% CI: 1.117–4.999, *p* = 0.0246) was a risk factor for less favorable PFS than grade III glioma (Table 2). Risk factor analysis for OS revealed that a pathology grading of IV (HR: 4.256, 95% CI: 1.487–12.182, *p* = 0.0070) and infratentorial tumor location (HR: 11.038, 95% CI: 1.309–93.083, *p* = 0.0273) were associated with reduced OS, whereas gross total resection (HR: 0.258, 95% CI: 0.120–0.554, *p* = 0.0005) and adjuvant radiotherapy (HR: 0.128, 95% CI: 0.031–0.524, *p* = 0.0043) were associated with improved OS. Neither scalp block (HR: 0.492, 95% CI: 0.222–1.089, *p* = 0.0800) nor *IDH1* mutation (HR: 0.728, 95% CI: 0.225–2.357, *p* = 0.5965) were associated with OS (Table 3).

**Table 2:** Risk factors of worse progression-free survival.

Factors	Univariate analysis			Multivariate analysis		
	HR	95% CI	p value	HR	95% CI	p value
Age	1.008	0.993–1.024	0.3067	0.990	0.971–1.010	0.3408
Sex (male: female)	1.393	0.871–2.229	0.1661	1.169	0.680–2.001	0.5718
Body mass index	1.006	0.948–1.056	0.9833			
Grading (IV: III)	2.126	1.155–3.914	0.0155	2.363	1.117–4.999	**0.0246**
Scalp block	0.467	0.286–0.762	0.0023	0.436	0.236–0.807	**0.0082**
Anesthesia (IV: IH)*	0.695	0.428–1.127	0.1401	0.749	0.408–1.377	0.3527
Red blood transfusion	0.722	0.359–1.454	0.3618	0.495	0.228–1.077	0.0764
Non-red blood transfusion	0.766	0.106–5.557	0.7918	0.563	0.071–4.478	0.5867
Gross total resection	0.441	0.268–0.726	0.0013	0.405	0.227–0.721	**0.0021**
Opioid consumption	1.009	0.999–1.003	0.3744	0.999	0.998–1.002	0.7488
Adjuvant radiotherapy	0.467	0.212–1.028	0.0586	0.420	0.103–1.716	0.2272
Adjuvant chemotherapy	0.769	0.381–1.552	0.4639	0.726	0.211–2.504	0.6126
**Surgeon**						
B: A C: A Others: A	1.389 1.237 0.895	0.789–2.447 0.615–2.490 0.376–2.128	0.2553 0.5508 0.8012	1.210 1.784 0.570	0.637–2.298 0.808–3.941 0.216–1.500	0.5598 0.1523 0.2547
Tumor location (infra: supra)	0.850	0.267–2.713	0.7843	1.187	0.333–4.238	0.7916
IDH1^R132H^ mutation	0.423	0.207–0.863	0.0180	0.304	0.118–0.784	**0.0138**

*IV* intravenous anesthesia, *IH* inhalational anesthesia, *supra* supratentorial, *Infra* infratentorial, *IDH* isocitrate dehydrogenase.

**Table 3:** Risk factors of worse overall survival.

Factor	Overall survival					
	Univariate analysis			Multivariate analysis		
	HR	95% CI	p value	HR	95% CI	p value
Age	1.001	0.979–1.023	0.9385	0.983	0.957–1.009	0.1912
Sex (male:female)	1.335	0.717–2.486	0.3621	0.969	0.462–2.032	0.9325
Body mass index	0.980	0.913–1.053	0.5892			
Grading (IV: III)	2.069	0.911–4.703	0.0825	4.256	1.487–12.182	**0.0070**
Scalp block	0.806	0.435–1.495	0.4938	0.492	0.222–1.089	0.0800
Anesthesia (IV: IH)	1.343	0.658–2.740	0.4182	1.432	0.569–3.606	0.4461
Red blood transfusion	0.885	0.372–2.109	0.7831	0.675	0.237–1.923	0.4614
Non-red blood transfusion	3.775	0.877–16.253	0.0745	5.718	0.961–34.029	0.0554
Gross total resection	0.331	0.163–0.672	0.0022	0.258	0.120–0.554	**0.0005**
Tumor location (infra: supra)	2.670	0.367–19.451	0.3324	11.038	1.309–93.083	**0.0273**
Opioid consumption	0.998	0.995–1.001	0.1338	0.997	0.994–1.001	0.0732
Adjuvant radiotherapy	0.155	0.066–0.362	< 0.0001	0.128	0.031–0.524	**0.0043**
Adjuvant chemotherapy	0.386	0.182–0.820	0.0133	0.686	0.188–2.507	0.5682
**Surgeon**						
B: A C: A Others: A	1.198 1.082 0.590	0.555–2.586 0.442–2.651 0.139–2.513	0.6452 0.8631 0.4757	0.891 1.606 0.272	0.379–2.096 0.588–4.382 0.058–1.267	0.7913 0.3552 0.0971
IDH1^R132H^ mutation	0.701	0.304–1.616	0.4043	0.728	0.225–2.357	0.5965

*IV* intravenous anesthesia, *IH* inhalational anesthesia, *supra* supratentorial, *infra* infratentorial, *IDH* isocitrate dehydrogenase.

The parallel analyses without overlapping included 39 patients and the results were summarized in the supplementary file (Supplementary Table 1 and Supplementary Table 2). In brief, the parallel multivariate Cox regression analysis also revealed that scalp block was associated with longer PFS. (HR: 0.095, 95% CI: 0.018–0.510, *p* = 0.0060; Supplementary Table 2).

## Discussion

The major discovery of this study was that scalp block is an independent prognostic factor for recurrence profile in patients with high-grade glioma, regardless of differences in *IDH1*^R132^^H^ mutation profile.

To the best of our knowledge, the major difference between previous reports and the present study is that presence of the *IDH1*^R132^^H^ mutation was for the first time included in an analysis of the influence of anesthetic technique on high-grade glioma oncological outcomes. A multigroup collaboration in 2008 sequenced 20,661 genes in 22 glioblastoma multiforme tumor samples and identified a common point mutation in the metabolic gene *IDH1* (R132H) that was present in 12% of patients with glioblastoma multiforme^19^. The proportion of *IDH1*^R132H^ in the present study (15.2%) is comparable to that reported previously. The study results are also compatible with those of previous studies indicating that patients with glioma carrying the *IDH1*^R132H^ mutation were younger and had longer PFS^12,20^. IDH1 is a key enzyme in Krebs cycle functions dependent on NADP^+^. Wild-type *IDH1* messenger RNA and protein are commonly overexpressed in primary glioblastomas, which indicates that *IDH1* activity is important to metabolic adaptation of high-grade gliomas^21^. In addition, IDH1 regulates hypoxia-inducible factors related to tumor angiogenesis and invasiveness^22^. This is relevant to glioma recurrence because glioma cells exhibiting invasive characteristics after resection surgery are more highly competitive for nutrients than are normal neuronal tissue. The beneficial outcomes observed among *IDH1*^R132H^ mutant gliomas may occur through several mechanisms. First, *IDH1*^R132H^ mutations result in neomorphic enzyme activity, catalyzing the NADPH-dependent reduction of α-ketoglutarate to *R*(-)-2-hydroxyglutarate^23^, which increases DNA methylation of the glioma. Data from a large cohort of 1,122 diffuse grade II–IV gliomas revealed that IDH-mutant gliomas with high levels of DNA methylation had more favorable clinical outcomes than those with low levels^24^. Second, *IDH1*^R132H^ mutations inhibit glial cell proliferation through inhibition of Bcl-xL, which induces more apoptosis than wild-type IDH^25^. Third, *IDH1*^R132H^ mitigates expression of hypoxia-induced factor-1α, which interferes with glioma angiogenesis^22^. Although the present study indicated improved PFS among patients carrying *IDH1*^R132H^, improved OS was not observed. This may be because the survival of patients with high-grade glioma has markedly improved in the last decade owing to a rapid increase in the use of adjuvant and concomitant chemotherapy and radiotherapy, earlier diagnosis, and advances in multimodal health care^26^. In addition, survival is influenced by more complex factors such as age, baseline performance status^27^, long-term care capability^28^, and gross total resection rate^29^. Therefore, the influence of *IDH1*^R132H^ on OS may have been obscured in our study.

The potential mechanisms of the protective effects of scalp blocks against high grade glioma recurrence are organized into three aspects, namely the prevention of a surgical stress response, the systemic effects of local anesthetic, and the sparing effects of general anesthetic. Accumulating evidence reveals that surgical stress and the subsequent neuroendocrine and inflammatory responses may negatively effects tumor recurrence after curative surgery^1^. First, the surgical stress response activates the sympathetic nervous system and the hypothalamic–pituitary–adrenal axis, thus promoting tumor-associated angiogenesis^30,31^. Second, surgical stress increases catecholamine circulation, which may impair immune function through mechanisms including diminished cytotoxicity of natural killer (NK) cells^32^, reduced dendritic cell maturity, and a decreased Th1/Th2 ratio, which suppresses antimetastatic cell-mediated immunity^33^. Furthermore, glioma cells express beta-adrenergic receptors, and catecholamine may thus interact with the glioblastoma proliferation^34^. Because scalp blocks effectively reduce surgical neuroendocrine stress, with concomitant reductions in plasma cortisol and catecholamine levels during craniotomy^35^, the aforementioned negative effects may be attenuated. In addition to the attenuation effects against stress responses, local anesthetics may prompt systemic protective effects against glioma proliferation through various mechanisms. First, local anesthetic blocks *N*-methyl-d-aspartate-type glutamate receptors from mediating rapid excitatory neurotransmission in the central nervous system^36,37^. Extracellular glutamate in the cerebral cortex negates *IDH1*^R132^^H^ suppression of gliomagenesis, thereby driving anchorage-independent growth and glioma progression^38^. Accordingly, negative effects of glutamate may be inhibited by the local anesthetics in scalp blocks. Second, transient receptor potential (TRP) channels regulate cell proliferation and death^39^. Local anesthetic (e.g., lidocaine) upregulates the TRPV1 channels and suppresses the TRPM7 channels, and both of these mechanisms protect against glioma cell proliferation^40,41^. Third, local anesthetic was reported to induce protective autophagy in a rat C6 glioma cell line^42^. Moreover, it weakened ZDHHC15 transcripts and reduced GP130 palmitoylation levels and membrane localization, thus impairing the growth and self-renewal of glioblastoma stem cells^43^.

General anesthetic agents may elicit multiple negative effects during the surgical resection of glioma. First, inhalational anesthetics were reported to profoundly suppress the effects of NK cells. A clinical study reported that sevoflurane induced a significant decrease in NK cells during cranial surgery^44^. Sevoflurane was also reported to attenuate NK cell–mediated cytotoxicity by inhibiting adhesion molecule leukocyte function-associated antigen-1 in an experimental study^45^. The importance of NK cells for glioma outcomes has been recently addressed; the *IDH1*^R132^ mutation promotes recruitment of NK cells in the brain and is associated with improved glioma prognosis^46^. In addition, novel therapies based on augmentation of NK cell recruitment and function are emerging in neuro-oncology^47^. Therefore, the negative effects of inhalational anesthetics on NK cells may lead to an immunosuppressant tumor microenvironment in patients undergoing glioma resection surgery. Second, the inhalational anesthetics may induce the negative gene expression effects of human glioma cells. Isoflurane and sevoflurane were reported to increase the migration, invasion, and colony-forming ability of human glioblastoma cells in vitro, and their tumor volume and invasion/migration were increased in vivo through increases in the expression of cell surface protein 44^48^. Third, both inhalational anesthesia and propofol were reported to increase the amount of circulating extracellular glutamate in patients undergoing neurosurgery^49^, which may promote glioma proliferation. Therefore, scalp blocks may attenuate the aforementioned negative effects through strong anesthetic-sparing effects^50^.

Intravenous anesthesia has also been proposed to result in less intraoperative immunosuppression than inhalational anesthesia during surgery^1^, and a recent meta-analysis revealed beneficial oncological outcomes of intravenous anesthesia in patients with nonglioma cancer. However, the current literature on glioma is limited and reveals nonsignificant effects^18,51,52^. In the present study, we observed comparably nonsignificant effects of intravenous anesthesia on oncological outcomes of patients with high-grade glioma. This may have been because of the unique characteristics of the glioma immune microenvironment. First, glioma cells may express cell surface proteins to sequester T cells in the brain^53^. Second, the blood–brain barrier acts as a physical barrier against the exchange of immune cells from extraneural tissues and the brain^54^. Third, natural killer cells are the least abundant immune cell infiltrating glioma cells (representing only 2.11% of all infiltrating immune cells^55^; and glioma cells secrete soluble immunosuppressive factors to suppress natural killer cell activity^56^. Because the protective immune effects of intravenous anesthesia are mainly exerted in the peripheral circulation, particularly through the activation of natural killer cells^57,58^, intravenous anesthesia may be less influential on the oncological outcomes of patients with glioma.

There are several limitations in this study. First, the study is single-centered, retrospective in nature, and with a relatively small sample. Second, despite the *IDH1*^R132^^H^ mutation being the most influential genetic factor for postoperative oncological outcomes of gliomas, information concerning other potential factors such as the chromosomal 1p/19q codeletion, *IDH2* or *MGMT* mutation, and *ATRX* mutation was not considered. Third, the number of patients with infratentorial tumors was much lower than those with supratentorial tumors. Hence, caution is needed when extrapolating the results to patients with infratentorial tumors. Fourth, steroids are not administered for patients with primary glioma in our institute; thus, the present results should be interpreted cautiously for patients receiving perioperative steroids.

In conclusion, our results indicate that scalp block is associated with favorable recurrence profiles in patients with high-grade glioma. However, given the limitations of this study and the complexity of the pathophysiology of glioma recurrence, our results should be considered exploratory rather than as a basis for practice change.

## Supplementary Information

Supplementary Tables.

## Data Availability

Datasets are available on request.
