# Supplementary material for: Influence of scalp block on oncological outcomes of high-grade glioma in adult patients with and without isocitrate dehydrogenase-1 mutation
Source: Sci Rep. 2021 Aug 13;11:16489. doi: 10.1038/s41598-021-95851-5 (PMC8363618; doi:10.1038/s41598-021-95851-5)
Supplement: Supplementary file 1 — Supplementary Tables. [file 41598_2021_95851_MOESM1_ESM.docx]

**Supplement Table 1. Characteristics of patients who received scalp block and those who did not receive scalp block**

|  | No Scalp block  (N=23) | Scalp block  (N=16) | p value |
| --- | --- | --- | --- |
| Age(years), mean ± SD | 55.5 ± 16.6 | 54.5 ± 13.3 | 0.1104 |
| Male (%) | 16 (69.6) | 8 (50.0) | 0.3178 |
| Body Mass Index (Kg/m^2^), mean ± SD | 23.64 ± 2.89 | 27.07 ± 4.89 | 0.0090 |
| Co-morbidities (%)  Cardiovascular  Hypertension  Diabetes mellitus  Liver and renal diseases | 4 (17.4)  6 (26.1)  3 (13.0)  1 (4.3) | 4 (25.0)  3 (18.8)  0 (0.0)  0 (0.0) | 0.6937  0.7110  0.2550  0.9999 |
| ASA class (%)  I  II  III  IV | 1 (4.3)  15 (65.2)  6 (26.1)  1 (4.3) | 1 (6.2)  10 (62.5)  4 (25.0)  1 (6.2) | 0.9855 |
| WHO grading (%)  III  IV | 2 (8.7)  21 (91.3) | 4 (25.0)  12 (75.0) | 0.2054 |
| Infratentorial tumor location (%) | 3 (13.0) | 1 (6.2) | 0.6309 |
| Intravenous anesthesia (%) | 17 (73.9) | 15 (93.7) | 0.2055 |
| Blood loss (ml),  median (Interquartile range) | 200  (100-500) | 200  (100-400) | 0.7651 |
| Blood transfusion, Red Blood(%) | 5 (21.7) | 0 (0.0) | 0.0660 |
| Blood transfusion, Non-Red blood (%) | 0 (0.0) | 0 (0.0) |  |
| Fentanyl usage (mcg),  median (Interquartile range) | 200.0  (156.3-287.5) | 225.0  (150.0-300.0) | 0.7728 |
| Gross total resection (%) | 3 (13.0) | 4 (25.0) | 0.4151 |
| Anesthesia duration(min),  median (Interquartile range) | 275.0  (203.0-313.8) | 284.5  (267.5-378.0) | 0.2359 |
| Surgeon (%)  A  B  C  D | 11 (47.8)  7 (30.4)  0 (0.0)  5 (21.7) | 9 (56.2)  3 (18.8)  0 (0.0)  4 (25.0) | 0.7130 |
| Adjuvant temozolamide (%) | 20 (87.0) | 13 (81.2) | 0.6743 |
| Adjuvant Radiotherapy (%) | 21 (91.3) | 15 (93.7) | 0.9999 |
| IDH-R132H mutation (%) | 1 (4.3) | 3 (18.8) | 0.2864 |

Abbreviation: ASA: American Society of Anesthesiologists; WHO: World Health Organization; IDH mutation: Isohydrate Dehydrogenase

| Supplement Table2. Risk factors of worse progression-free survival | | | | | | |
| --- | --- | --- | --- | --- | --- | --- |
| Factors | **Univariate analysis** | | | **Multivariate analysis** | | |
|  | **HR** | **95% CI** | **p Value** | **HR** | **95% CI** | **p-Value** |
| Age | 0.986 | 0.961-1.012 | 0.2962 | 0.990 | 0.957-1.025 | 0.5731 |
| Sex  (Male: Female) | 0.974 | 0.423-2.243 | 0.9500 | 1.633 | 0.483-5.522 | 0.4300 |
| Body Mass Index | 0.867 | 0.765-0.982 | 0.0246 |  |  |  |
| Grading  (IV: III) | 1.864 | 0.551-6.305 | 0.3168 | 2.191 | 0.432-11.11 | 0.3439 |
| Scalp block | 0.445 | 0.181-1.099 | 0.0793 | 0.095 | 0.018-0.510 | 0.0060 |
| Anesthesia  (IV: IH)* | 0.666 | 0.219-2.031 | 0.4752 | 0.916 | 0.204-4.117 | 0.9088 |
| Transfusion^#^ | 0.212 | 0.029-1.581 | 0.1302 | 0.014 | 0.001-0.267 | 0.0044 |
| Gross total resection | 0.271 | 0.063-1.160 | 0.0784 | 0.724 | 0.122-4.299 | 0.7225 |
| Opioid consumption | 1.001 | 0.997-1.006 | 0.6478 | 1.004 | 0.998-1.010 | 0.2108 |
| Adjuvant  radiotherapy | 104468 | 0-1.89x10^252^ | 0.9683 | 37160 | 0-32.7x10^258^ | 0.9657 |
| Adjuvant  Chemotherapy | 0.794 | 0.180-3.500 | 0.7606 | 0.234 | 0.026-2.187 | 0.2054 |
| Surgeon^&^  B: A  D: A | 1.093  0.708 | 0.423-2.826  0.244-2.057 | 0.8547  0.5254 | 1.826  0.740 | 0.451-7.390  0.187-2.292 | 0.3986  0.6671 |
| Tumor location  (Infra: Supra) ^#^ | 1.240 | 0.290-5.299 | 0.7716 | 1.321 | 0.210-8.311 | 0.7671 |
| IDH-R132H  Mutation | 1.916 | 0.641-5.732 | 0.2446 | 5.898 | 0.677-51.42 | 0.1082 |

Abbreviation: *IV: Intravenous anesthesia; IH: Inhalational anesthesia; ^#^ Supra: supratentorial, Infra: infratentorial

^#^ In cases included in parallel analysis, no non-red blood cell transfusion is performed.

^&^ In cases included in parallel analysis, no patient was treated in Surgeon C’s category.
